# Coal Oil Poisoning

**Published:** 1892-12

**Authors:** W. H. Ridge


### COAL OIL POISONING.

By W. H. RIDGE, V.M D.

I was called to attend a cow on November 11th. The animal got out of the stable the night before, getting into a neighbor's yard, the neighbor having five gallons of coal oil in a wash boiler, the boiler being buried in the sand to prevent it from being upset. The can was covered by a board. The cow pushed the board off, and drank a little over three gallons.

*Symptoms*—The next forenoon she stood with back arched, tympanitic, head extended, mouth open, tears flowing down the face, discharge of frothy fluid from mouth and nose which smelled strongly of petroleum The animal was trembling all over, looked as if extremely nauseated. The horns, legs and ears were cold. Could not find any pulse, heart beat weak, respiration frequent, but irregular. Staggered when moved, kept shaking the head up and down.

Gave Sodii Sulph. 1 lb. at once. Then gave

*Receipe.* Ext. Dig. Fl. ½ oz.
Cinchonæ Sulph. 1 oz.
Spt. Vini Rect.
Aqua Font. aa 2 oz.
Ac. Sulphuric 4 drms.
*Misce.*
*Sig.*: Tablespoonful every two hours.

The next morning seemed much better; did not tremble, walked around stronger, mouth dry, pulse irregular, heart-beat stronger. Toward night commenced with symptoms of meningitis (from the description of owner). Soon got down, and was unable to rise again, dying in about three hours after getting down. The points to be noted are the length of time she lived after drinking such a large amount.

### A PLEA FOR THE MORE THOROUGH STUDY OF THE STRUCTURE OF THE HORSE.

By R. W. Shufeldt, Medical Department U. S. Army.

It is quite remarkable to me sometimes what a lack of knowledge exists, even among those supposed to be well-informed, of equine anatomy. It is difficult to see why this should be the case, for the literature of the subject is by no means meagre, and the opportunities to dissect horses are ample. Not long ago a gentleman in Chicago, a perfect stranger to the writer, wrote me that he had secured a most remarkable fossil bone of great size in the excavations being made at the World's Fair Buildings. He had shown it to many distinguished veterinarians in Chicago, all of whom could give him no information as to what kind of an animal it had belonged. Even the President of the Academy of Sciences, of Chicago, stated to him after carefully examining the bone, that no doubt it was from a fossil rhinoceros! The specimen was duly sent on to me for identification, and I pronounced it the *right femur* of a modern horse, as soon as I set my eyes upon it. My Chicago correspondent wrote me that it was from the glacial drift, but I found the bone by no means a fossil, though it had the appearance of having lain long in some gravel or clay bed.

Numerous instances of this kind have fallen to my personal experience during the last twenty years or more, and they have always excited their due measure of surprise. The question absolutely bristles with the most cogent reasons why the anatomy of the horse should be most exhaustively studied, and assuredly by those whose profession it is to know it. Horses stand among the most valued of all our domestic animals and they cannot be scientificly treated when diseased, nor bred to advantage without our being in possession of a knowledge of their entire structure. A thorough understanding of the anatomy of a horse is of the very greatest importance, too, to the comparative morphologist, and a knowledge of the osteology of the animal quite indispensable to the working paleontologist. With the rarest exception the government veterinarian in the army of this country knows little or nothing of the subject of equine anatomy, and this stricture may with equal truth be extended to include the West Point graduates in the artillery and cavalry arms of the service. After many years service on the active list of our regular army, I never met with a single U. S. officer of cavalry who knew enough of the anatomy of a horse as to be able to enumerate one-third of the bones of its skeleton correctly. Our general government is chiefly at fault here, and the subject of veterinary science should not only receive more attention at West Point, but we should also have in this country at least one first-class Veterinary College for the instruction of government veterinarians. It is probably beyond all hope that the Army Medical Museum at Washington can ever give any special assistance in this matter, notwithstanding the fact that it could easily do so, and then hardly be considered as having passed beyond the confines of its proper province. To those most interested it would seem quite in order to bring a bill before the coming Congress to establish a National School of Veterinary Science at Washington, to be supported by the government and to graduate thoroughly educated veterinarians for the government service.

